# Non-invasive biomarkers prognostic of decompensation events in NASH cirrhosis: a systematic literature review

**DOI:** 10.1007/s00109-024-02448-2

**Published:** 2024-05-16

**Authors:** Mattia Amoroso, Salvador Augustin, Sven Moosmang, Isabella Gashaw

**Affiliations:** 1 Boehringer Ingelheim Pharma GmbH, Biberach, Germany; 2 Boehringer Ingelheim International GmbH, Ingelheim, Germany; 3 Boehringer Ingelheim Pharma GmbH, Ingelheim, Germany

**Keywords:** Liver stiffness, Type 2 diabetes, Hyaluronic acid, fibrosis, imaging, liver, nonalcoholic steatohepatitis, predict, serum

## Abstract

Liver cirrhosis due to nonalcoholic steatohepatitis (NASH) is a life-threatening condition with increasing incidence world-wide. Although its symptoms are unspecific, it can lead to decompensation events such as ascites, hepatic encephalopathy, variceal hemorrhage, and hepatocellular carcinoma (HCC). In addition, an increased risk for cardiovascular events has been demonstrated in patients with NASH. Pharmacological treatments for NASH cirrhosis are not yet available, one of the reasons being the lack in surrogate endpoints available in clinical trials of NASH cirrhosis. The feasibility of non-invasive prognostic biomarkers makes them interesting candidates as possible surrogate endpoints if their change following treatment would result in better outcomes for patients in future clinical trials of NASH cirrhosis. In this systematic literature review, a summary of the available literature on the prognostic performance of non-invasive biomarkers in terms of cardiovascular events, liver-related events, and mortality is outlined. Due to the scarcity of data specific for NASH cirrhosis, this review includes studies on NAFLD whose evaluation focuses on cirrhosis. Our search strategy identified the following non-invasive biomarkers with prognostic value in studies of NASH patients: NAFLD fibrosis score (NFS), Fibrosis-4 (FIB-4), aspartate aminotransferase (AST) to platelet ratio index (APRI), enhanced liver fibrosis (ELF™), BARD (BMI, AST/ALT (alanine aminotransferase) ratio, diabetes), Hepamet Fibrosis Score (HFS), liver enzymes (AST + ALT), alpha-fetoprotein, platelet count, neutrophil to lymphocyte ratio (NLR), Lysyl oxidase-like (LOXL) 2, miR-122, liver stiffness, MEFIB (liver stiffness measured with magnetic resonance elastography (MRE) + FIB-4), and PNPLA3 GG genotype. The aim of the present systematic literature review is to provide the reader with a summary of the non-invasive biomarkers with prognostic value in NASH cirrhosis and give an evaluation of their utility as treatment monitoring biomarkers in future clinical trials.

## Introduction

Nonalcoholic steatohepatitis (NASH) is characterized histologically by the concomitant presence of liver steatosis, hepatocellular injury (hepatocyte ballooning), and lobular inflammation. These factors can lead to varying degrees of fibrosis deposition, ranging from mild to cirrhosis [1, 2]. Hepatic clinical decompensation events of cirrhosis such as ascites, hepatic encephalopathy, variceal hemorrhage, and hepatocellular carcinoma (HCC) [3–5] can be life-threatening and significantly impact patients’ quality of life [6, 7]. The presence of bridging fibrosis (F3) and cirrhosis (F4) serves as an important marker for liver-related outcomes and overall mortality prognosis [8–12]. Although liver biopsy is considered the gold standard for diagnosing and staging liver fibrosis [13], it has well-known limitations, including invasiveness, poor acceptability, high costs, sampling variability and inter-observer variability [13, 14]. Notably, patients with advanced fibrosis are at higher risk to progress to decompensation events, portal hypertension or death [3–5, 15–20]. In addition to increasing the risk of developing liver-related events (LREs) such as ascites, hepatic encephalopathy, variceal hemorrhage, and hepatocellular carcinoma, advanced fibrosis is an independent predictor of cardiovascular events (CVEs) such as stroke, myocardial infarction, coronary revascularization, and cardiac-related death [21]. Despite the burden that decompensated NASH cirrhosis imposes on patients as well as health systems worldwide [22], no specific pharmacological treatment is currently available. A reason for this relies partly on the fact that no surrogate endpoint is yet available in clinical studies of NASH cirrhosis. Hence, clinical development for NASH cirrhosis is lengthy with little possibilities to focus on promising drug candidates based on the results of earlier stage clinical trials and requires outcome-driven pivotal trials. To support the development of future treatment concepts targeting NASH cirrhosis, biomarkers with prognostic value can be considered for treatment monitoring in late-stage clinical development (phase IIb-III). According to the indications proposed by the FDA-NIH Biomarker Working Group and summarized in the BEST (Biomarkers, EndpointS, and other Tools) guidelines, prognostic biomarkers are used to identify the likelihood for a clinical event, disease recurrence or progression to occur in patients with the medical condition of interest [23]. Non-invasive biomarkers are desirable in clinical trials, being generally simple to measure and clinically feasible for longitudinal testing. Identifying reliable prognostic biomarkers would aid clinical development if treatment-related changes in these biomarkers would correlate with better outcomes. Since an approved treatment for NASH is still lacking, the focus is currently on prognostic biomarkers. Proving their monitoring capabilities will be a subsequent step when more clinical outcome data becomes available.

The aim of this systematic literature review is to summarize the evidence on non-invasive prognostic biomarkers predictive of decompensation, LREs, and/or CVEs available in the literature to date. Data from retrospective/longitudinal studies are presented and discussed, with particular emphasis on the role of such biomarkers in predicting all-cause mortality, LREs, and HCC occurrence in NASH patients with bridging fibrosis and cirrhosis. Finally, for each biomarker and based on the information collected in the presented studies, we attempt to evaluate their potential utility as treatment monitoring biomarkers in future clinical trials.

## Methods

### Search strategy

To find potentially suitable studies, a comprehensive search strategy was implemented using the OVID database [24]. This approach included searching for relevant terms within the title, abstract, or text words throughout the record, as well as in the medical subject heading (MeSH). Following this, the titles, abstracts, and full texts of the identified studies were retrieved and assessed against previously specified inclusion and exclusion criteria. The PRISMA principles guided the preparation of the present review [25] (Table S1).

### Inclusion and exclusion criteria

**Fig. 1:**
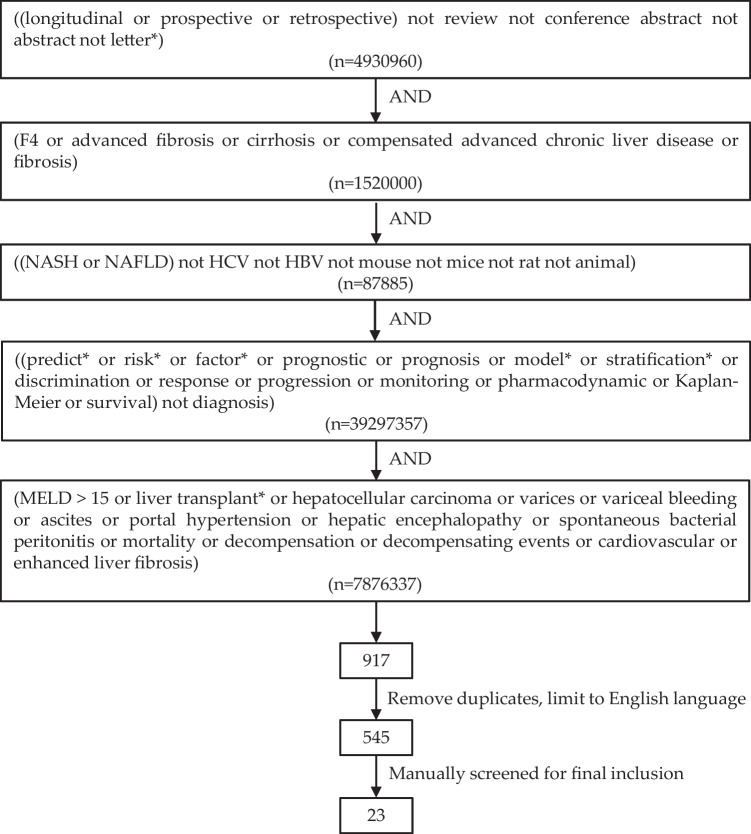
Flow diagram of search strategy in OVID database for the included studies including results (date of data retrieval: 3 January 2023)

A search was conducted for studies published in peer-reviewed journals that evaluated the prognostic accuracy of at least one non-invasive biomarker of interest (refer to Tables 1 and 2 for a complete list) in predicting CVEs, LREs, or mortality. The search terms for the events of interest included model for end-stage liver disease (MELD) score > 15, liver transplant, hepatocellular carcinoma, varices or variceal bleeding, ascites, portal hypertension, hepatic encephalopathy, spontaneous bacterial peritonitis, mortality (liver-related or all-cause), decompensation events, cardiovascular events, enhanced liver fibrosis. Only publications in English were considered for inclusion. Review articles, conference abstracts, letters to editors, and in general studies involving non-human animal models were excluded. Data on patients with liver diseases other than NASH (e.g., HCV, HBV, autoimmune hepatitis) were not considered. Only articles that included patients with F3-F4 fibrosis at baseline were considered. Studies with patients at different fibrosis stages at baseline were included only if outcomes for the F3-F4 population were reported. In order to highlight data relevant to the F3-F4 population in studies involving mixed cohorts, Tables 1 and 2 also include the relative percentage of patients with advanced fibrosis at baseline, defined as having liver fibrosis of stage ≥ F3. Following deduplication of redundant results, the search strategy yielded 545 results, which were manually screened for relevance. Ultimately, 23 studies published between 2013 and 2022 were included in this systematic review. A flow diagram summarizing the search strategy is outlined in Fig. 1.

**Table 1:** Non-invasive biomarkers prognostic of cardiovascular events

**Biomarker**	***N***	**Patients with advanced fibrosis at baseline (%)**	**Ethnicity**	**Prognostic score**	**Median follow-up**	**Outcomes predicted**	**Ref.**
**NFS**	660	NA	NA	> 0.676	41.4 months	CVEs (HR: 2.29, 95% CI: 1.17–4.47)	[27]
	285	42.3	Caucasian, Hispanic, Black, Asian	> 0.676	5.2 years	CVEs (HR: 4.61, 95% CI: 2.28–9.32)	[21]
	11,154	NA	Caucasian, Black, Hispanic	> 0.676	14.5 years	Mortality due to CVEs (HR: 3.46, 95% CI: 1.91–6.25)	[28]
	608	24.1	Caucasian	NA	81 months	CVEs (Harrel’s c-index = 0.648 ± 0.0394)	[35]
**FIB-4**	660	NA	NA	> 2.67	41.4 months	CVEs (HR: 4.57, 95% CI: 1.61–12.98)	[27]
	11,154	NA	Caucasian, Black, Hispanic	1.30–2.67 > 2.67	14.5 years	FIB-4 1.30–2.67 predicts mortality due to CVEs (HR: 1.75, 95% CI: 1.26–2.43) FIB-4 > 2.67 predicts mortality due to CVEs (HR: 2.68, 95% CI: 1.44–4.99)	[28]
	608	24.1	Caucasian	NA	81 months	CVEs (Harrel’s c-index = 0.6 ± 0.0253)	[35]
**APRI**	11,154	NA	Caucasian, Black, Hispanic	> 1.5	14.5 years	CVEs (HR: 2.53, 95% CI: 1.33–4.83)	[28]
**BARD**	608	24.1	Caucasian	NA	81 months	CVEs (Harrel’s c-index = 0.644 ± 0.0442)	[35]
**LSM**	105	35.2	Caucasian, Hispanic	≥ 2.97 kPa (MRE)	19 months	CAC (OR: 3.53, 95% CI: 1.29–10.48)	[94]

*APRI* AST to platelet ratio index, *BARD BMI AST/ALT* ratio, diabetes, *CAC* coronary artery calcification, *CI* confidence interval, *CVEs* cardiovascular events, *FIB*-*4* fibrosis-4, *HR* hazards ratio, *LSM* liver stiffness measurement, *MRE* magnetic resonance elastography, *NA* not available, *NFS* NAFLD fibrosis score, *OR* odds ratio

**Table 2:** Non-invasive biomarkers prognostic of liver-related events

**Biomarker**	***N***	**Patients with advanced fibrosis at baseline (%)**	**Ethnicity**	**Prognostic value (baseline)**	**Median follow-up**	**Outcomes predicted**	**Ref.**
**NFS**	320	51	Caucasian, Asian, Black, Native American	> 0.676	104.8 months	LREs (AUC = 0.86, 95% CI: 0.80–0.92. HR: 34.2, 95% CI: 6.5–180.9); overall mortality (AUC = 0.70, 95% CI: 0.62–0.78. HR: 9.8, 95% CI: 2.7–35.3)	[31]
	11,154	NA	Caucasian, Black, Hispanic	> 0.676	14.5 years	Overall mortality (HR: 1.69, 95% CI: 1.09–2.63)	[28]
	148	33.8	NA	> 0.676	5 years	LREs (AUROC = 0.79, 95% CI: 0.69–0.91. Sensitivity: 50; specificity: 90.1; PPV: 47; NPV: 91.1; HR: 11.9, 95% CI: 3.79–37.4)	[30]
	153	20.9	NA	> 0.676	100 months (mean)	Overall mortality (AUC = 0.8044; HR: 1.58, 95% CI: 1.23–1.88), occurrence of malignancies (HR: 1.27, 95% CI: 1.05–1.42), LREs (HR: 5.12, 95% CI: 2.62–10.01), admissions (HR: 1.74, 95% CI: 1.31–2.31) and duration of hospitalization (HR: 1.61, 95% CI: 1.23–2.10)	[29]
	180	Not specified	Asian (Chinese)	> − 1.455 > − 1.836	6.6 years	NFS > − 1.455: Overall mortality (HR: 2.74, 95% CI: 1.67–4.50; AUROC = 0.828, 95% CI: 0.73–0.93) NFS > − 1.836: Overall mortality (sensitivity: 88.3%; specificity: 61.9%)	[33]
	446	32.3	Asian (Japanese)	> -1.455	4.6 years	Overall mortality (HR: 12.87, 95% CI: 1.35–122.30)	[32]
	4163	NA	Asian (Korean)	≥ − 2.08	15.6 years	Overall mortality (AUROC = 0.67, 95% CI: 0.64–0.69) (HR: 1.43, 95% CI: 1.21–1.68; sensitivity: 70.0%; specificity: 55.3%)	[34]
	258	100	Caucasian, Hispanic	NA	30.9 months	LREs (HR: 1.78, 95% CI: 1.43–2.21)	[11]
	608	24.1	Caucasian	NA	81 months	LREs (Harrel’s c-index = 0.796 ± 0.0231), HCC (Harrel’s c index = 0.901 ± 0.0302)	[35]
**FIB-4**	148	33.8	NA	> 3.25	5 years	LREs (AUROC = 0.89, 95% CI: 0.83–0.95; sensitivity: 56.3; specificity: 91.2; PPV: 52.9; NPV: 92.2. HR: 6.33, 95% CI: 1.98–20.2)	[30]
	442,227	21.7	Caucasian, Black, Hispanic, native American	≥ 2.67 1.30–2.66	34.8 months	FIB-4 ≥ 2.67: overall mortality (HR: 2.49, 95% CI: 2.20–2.82), end-stage liver disease (HR: 1.86, 95% CI: 1.68–2.05), development of HCC (HR: 3.66, 95% CI: 2.71–4.94), liver transplantation (HR: 7.98, 95% CI: 4.62–13.79) FIB-4 1.3–2.66: overall mortality (HR: 1.13, 95% CI: 1.02–1.26), end-stage liver disease (HR: 1.14, 95% CI: 1.07–1.22)	[39]
	320	51	Caucasian, Asian, Black, Native American	> 2.67	104.8 months	LREs (AUC = 0.81, 95% CI: 0.76–0.87; HR: 14.6, 95% CI: 4.1–52.6), overall mortality (AUC = 0.67, 95% CI: 0.58–0.76; HR: 6.9, 95% CI: 2.3–20.4)	[31]
	153	20.9	NA	> 2.67	100 months (mean)	Overall mortality (HR: 10.52, 95% CI: 2.98–37.07), occurrence of malignancies (HR: 6.12, 95% CI: 2.31–16.17), LREs (HR: 13.05, 95% CI: 5.78–31.54), admissions (HR: 3.80, 95% CI: 2.79–5.19) and duration of hospitalization (HR: 2.69, 95% CI: 1.92–3.78)	[29]
	365	29.8	Asian (Japanese)	≥ 2.67	7.1 years	Lower survival (67.3% survival at 10 years vs. 96.4% in FIB-4 < 2.67), higher incidence of HCC (15% at 10 years vs. 0.5% in FIB-4 < 2.67)	[63]
	996	38.1	NA	≥ 1.30	2.5 years	Development of HCC (HR: 8.46, 95% CI: 1.06–67.37)	[40]
	11,154	NA	Caucasian, Black, Hispanic	1.30–2.67	14.5 years	Overall mortality (HR: 1.46, 95% CI: 1.16–1.82)	[28]
	4163	NA	Asian (Korean)	≥ 1.22	15.6 years	Overall mortality (AUROC = 0.69, 95% CI: 0.66–0.71; HR: 1.41, 95% CI: 1.18–1.68; sensitivity: 64.4%; specificity: 64.6%)	[34]
	608	24.1	Caucasian	NA	81 months	LREs (Harrel’s c-index = 0.783 ± 0.0288), overall mortality (Harrel’s c index = 0.850 ± 0.0135)	[35]
	475	100	Caucasian, Hispanic	NA	30.9 months	LREs (HR: 1.24, 95% CI: 1.14–1.35)	[11]
**APRI**	153	20.9	NA	> 1.5	100 months (mean)	Occurrence of malignancies (HR: 4.94, 95% CI: 1.92–12.82), LREs (HR: 6.55, 95% CI: 3.13–13.72), admissions (HR: 2.49, 95% CI: 1.80–3.43) and duration of hospitalization (HR: 2.90, 95% CI: 2.11–3.98)	[29]
	320	51	Caucasian, Asian, Black, Native American	> 1.5	104.8 months	LREs (AUC = 0.80, 95% CI: 0.73–0.86; HR: 20.9, 95% CI: 2.6–165.3), overall mortality (AUC = 0.63; 95% CI: 0.53–0.72; HR: 3.1, 95% CI: 1.1–8.4)	[31]
	148	33.8	NA	> 1.5	5 years	LREs (AUROC = 0.89, 95% CI: 0.82–0.96; sensitivity: 50; specificity: 92.3; PPV: 50; NPV: 92.3; HR: 5.02, 95% CI: 1.6–15.7)	[30]
	475	100	Caucasian, Hispanic	NA	30.9 months	LREs (HR: 1.88, 95% CI: 1.45–2.46)	[11]
**BARD**	320	51	Caucasian, Asian, Black, Native American	4	104.8 months	LREs (AUC = 0.73, 95% CI: 0.66–0.80; HR: 6.6, 95% CI: 1.4–31.1), overall mortality (AUC = 0.66, 95% CI: 0.58–0.74)	[31]
	608	24.1	Caucasian	NA	81 months	LREs (Harrel’s c-index = 0.728 ± 0.0181), HCC (Harrel’s c index = 0.772 ± 0.0345), extrahepatic cancer (Harrel’s c-index = 0.624 ± 0.0442)	[35]
**ELF score**	475	100	Caucasian, Hispanic	≥ 11.27	30.9 months	LREs (HR: 2.11, 95% CI: 1.53–2.90; sensitivity: 51%; specificity: 72%)	[11]
**HFS**	608	24.1	Caucasian	NA	81 months	Overall mortality (Harrel’s c index = 0.849 ± 0.0187)	[35]
**Liver enzymes**	42,282	NA	Caucasian, Black, Hispanic	ALT > 40 IU/mL (men) ALT > 31 IU/mL (women)	8.4 years (median)	HCC development (HR: 4.35, 95% CI: 1.90–9.94)	[55]
	7068	100	Caucasian, Black, Hispanic	AST/√ALT > 6.45	3.7 years (mean)	HCC development (HR > 1.99)	[56]
**Alpha-fetoprotein**	351	31.3	Asian (Japanese)	≥ 5 μg/L	4.2 years	HCC development (HR: 7.15, 95% CI: 1.44–35.6)	[61]
**Platelet count**	2666	6.4	NA	< 150 × 10^3^/µL	60.7 months	HCC development (HR: 3.67, 95% CI: 1.95–10.40)	[62]
	7068	100	Caucasian, Black, Hispanic	< 146 × 10^3^/µL	3.7 years (mean)	HCC development (HR > 2.18)	[56]
	365	29.8	Asian (Japanese)	< 115 × 10^3^/μL	7.1 years	Overall mortality (48.8% vs. 91.2% survival at 10 years), liver-related mortality (62.2% vs. 94.2% survival at 10 years), HCC (20.6% vs. 4.4% occurrence at 10 years) vs. group with platelet count ≥ 11.5 × 10^4^/μL	[63]
**NLR**	9300	12.4	NA	≥ 3.09	5.5. years (mean)	HCC development (HR: 1.43, 95% CI: 1.01–2.03)	[70]
**Absolute lymphocyte count**	9280	12.4	NA	≥ 2.15	5.5 years (mean)	Lower HCC development (HR: 0.64, 95% CI: 0.43–0.94)	[70]
**sLOXL2**	475	100	Caucasian, Hispanic	Not specified	30.9 months	LREs (HR: 1.02, 95% CI: 1.01–2.04)	[11]
**miR-122 (serum)**	81	51.8	Asian (Japanese)	miR-122 ratio < 0.5	7.6 years	HCC development (range: 0.0–7.8 years), overall mortality (range: 0.5–7.9 years)	[87]
**LSM**	265	18	Caucasian, Hispanic	≥ 3.63–5 kPa ≥ 5 kPa (MRE)	≥ 8 years	≥ 3.63–5 kPa: primary outcomes (HR: 17.09, 95% CI: 2.38–122.75) ≥ 5 kPa: primary outcomes (HR: 16.58, 95% CI: 2.90–94.79)	[95]
	2666	6.4	Not specified	≥ 9.3 kPa (TE)	60.7 months	HCC development (HR: 13.76, 95% CI: 2.83–66.95)	[62]
	2245	Not specified	Caucasian, Asian	> 12 kPa (Fibroscan^®^)	27 months	Overall mortality (HR: 2.85, 95% CI: 1.65–4.92), increased 5-years incidence of death (13.8%) and liver events (10.2%)	[97]
	1398	100	Caucasian, Hispanic	≥ 30.7 kPa (Fibroscan^®^)	16.2 months	LREs (HR: 10.52, 95% CI: 5.15–21.48)	[98]
	128	26	Not specified	> 19% increase at follow-up from baseline (MRE)	7.7 years	Decompensation events and overall mortality (HR: 19.04, 95% CI: 3.11–117)	[96]
**MEFIB**	265	18	Caucasian, Hispanic	Positive MEFIB index (FIB-4 ≥ 1.6 + MRE ≥ 3.3 kPa)	≥ 8 years	A positive MEFIB index (combination of MRE ≥ 3.3 kPa and FIB-4 ≥ 1.6), had a strong association with prevalent and future incident liver-related outcomes or death. Each 1-kPa increase in liver stiffness was associated with 2.20-fold increased odds of prevalent hepatic decompensation or HCC	[95]
**PNPLA3 GG genotype**	238 (167 with NASH)	25.1	Asian (Japanese)	Presence of variant	6.1 years	HCC development (HR: 6.36, 95% CI: 1.36–29.8)	[103]

*ALT* alanine aminotransferase, *AST* aspartate aminotransferase, *APRI* AST to platelet ratio index, *BARD* *BMI AST/ALT* ratio type 2 diabetes, *CI* confidence interval, *ELF™* Enhanced Liver Fibrosis, *FIB-4* Fibrosis-4 index, *HCC* hepatocellular carcinoma, *HFS* Hepamet Fibrosis Score, *HR* hazard ratio, *LREs* liver-related events, *MEFIB* MRE + FIB-4, *LSM* liver stiffness measurement, *MRE* magnetic resonance elastography, *NA* not available, *NAFLD* nonalcoholic fatty liver disease, *NFS* NAFLD fibrosis score, *NLR* neutrophil to lymphocyte ratio, *PNPLA3* patatin-like phospholipase domain containing 3, *sLOXL2* serum lysyl oxidase-like 2

### Evaluation of treatment monitoring utility of prognostic non-invasive biomarkers

In this study, we assessed the utility of various prognostic non-invasive biomarkers for treatment monitoring in drug-interventional clinical trials for NASH cirrhosis. Each biomarker was evaluated based on its potential sensitivity to treatment and the invariability of its parameters. The biomarkers were categorized into three levels of utility for treatment monitoring: low, medium, and high. The reasons for their utility were identified, focusing on the nature of the parameters included in their formulas. For instance, biomarkers with formulas containing invariable parameters such as age or presence of diabetes were generally given a lower utility rating. Conversely, biomarkers whose formulas included parameters potentially sensitive to treatment, such as AST or platelet count, were given a higher utility rating. Additional comments were provided where necessary, highlighting specific characteristics of the biomarkers or the need for more treatment data. Established treatment monitoring biomarkers were identified, and their limitations were noted. The utility of each biomarker was then tabulated, providing a comprehensive overview of their potential use in treatment monitoring in the context of drug-interventional clinical trials.

## Results

### Non-invasive biomarkers with prognostic value

To ease the navigation through the sections of the present systematic review, each prognostic biomarker is categorized into four main sections: serum biomarkers, imaging biomarkers, combination of serum and imaging biomarkers, and genomic biomarkers. Moreover, the findings are presented based on the two key outcomes predicted by these biomarkers: CVEs (Table 1) and LREs (Table 2). If not otherwise stated, CVEs are defined as fatal or nonfatal ischemic stroke, myocardial infarction, cardiac or peripheral revascularization, arterial fibrillation, cardiovascular death, or a combination thereof. The same principle applies to LREs, which are defined as ascites, portosystemic encephalopathy, hepatopulmonary syndrome, spontaneous bacterial peritonitis, hepatorenal syndrome, or a combination thereof. Finally, for each biomarker, an evaluation of its potential utility as treatment monitoring biomarkers is summarized in Table 3.

**Table 3:** Treatment monitoring utility of prognostic non-invasive biomarkers

**Biomarker**	**Treatment monitoring utility**	**Reasons for its utility**	**Additional comments**
**NFS**	Low	Its formula contains invariable parameters (age)	The presence of diabetes or impaired fasting glucose increases its score
**FIB-4**	Medium	Its formula contains invariable parameters (age)	Treatment-related changes likely driven by liver enzymes
**APRI**	High	The parameters its formula contains are potentially sensitive to treatment (AST, platelet count)	Treatment-related changes likely driven by liver enzymes
**BARD**	Low	Its formula contains invariable parameters (presence of diabetes)	
**ELF**	High	The parameters its formula contains are potentially sensitive to treatment (HA, TIMP-1, PIIINP)	The range of the score is rather small
**HFS**	Low	Its formula contains invariable parameters (age, sex, diabetes)	
**Liver enzymes**	Medium	Established treatment monitoring biomarkers. ALT not always elevated in NASH patients	
**Alpha-fetoprotein**	Low	Rather diagnostic	
**Platelet count**	Medium	Won’t qualify as surrogate endpoint as stand-alone	More treatment data needed
**NRL**	Medium/high	Inflammatory parameter, it can be used as early marker of HCC	More treatment data needed
**Absolute lymphocyte count**	Medium/high	Inflammatory parameter, can be used as early marker of HCC	More treatment data needed
**sLOXL2**	Low	Inflammatory parameter	Difficult to establish as surrogate endpoint because of failure of anti-LOXL treatment
**miR-122**	Medium	Decrease over time predicts HCC	
**LSM**	High	Established treatment monitoring biomarker under controlled conditions	
**MEFIB**	High	Combination of imaging + serum marker	More data on its use as treatment monitoring biomarker needed

*ALT* alanine aminotransferase, *APRI* AST to platelet ratio index, *AST* aspartate aminotransferase, *BARD BMI* AST/ALT ratio type 2 diabetes, *ELF*™ enhanced liver fibrosis, *FIB*-*4* fibrosis-4 index, *HCC* hepatocellular carcinoma, *HFS* Hepamet Fibrosis Score, *LSM* liver stiffness measurement, *MEFIB* MRE + FIB-4, *MRE* magnetic resonance elastography, *NAFLD* nonalcoholic fatty liver disease, *NFS* NAFLD fibrosis score, *sLOXL2* serum lysyl oxidase-like 2

### Serum biomarkers

#### NFS

The NAFLD Fibrosis Score (NFS) is a non-invasive score that was initially developed and validated as a diagnostic tool by Angulo and colleagues to discriminate between the presence or absence of advanced fibrosis (F3-F4) in NAFLD patients [26]. Its formula includes age, body mass index (BMI), presence of impaired fasting glucose or diabetes mellitus, aspartate aminotransferase (AST)/alanine aminotransferase (ALT) ratio, platelet count, and serum albumin levels. In the current review, a total of nine studies identified NFS as prognostic biomarker of LREs and four of CVEs. Several threshold values are suggested for NFS to be prognostic of CVEs, LREs, and/or mortality. NFS > 0.676 is the most commonly used threshold and predicted CVEs occurring within 3.5–5.2 years follow-up (HR: 2.29–4.61, Table 1) [21, 27] as well as mortality due to CVEs over 14.5 years follow-up (HR: 3.46 (95% CI: 1.91–6.25), Table 1) [28]. In addition, NFS > 0.676 predicted LREs occurring within 5–8.7 years follow-up (HR: 5.12–34.20, Table 2) [29–31] as well as overall mortality occurring within 100–104.8 months follow-up (HR: 1.58–9.80, Table 2) [29, 31]. Furthermore, NFS > 0.676 predicted the increased occurrence of malignancies other than HCC (HR: 1.27 (95% CI: 1.05–1.42), Table 2), increased hospital admissions (HR: 1.74 (95% CI: 1.31–2.31), Table 2), and duration of hospitalization within 100 months (HR: 1.61 (95% CI: 1.23–2.10), Table 2) [29]. While the above-mentioned outcomes for NFS > 0.676 were predicted in cohorts of mixed ethnicities, lower NFS thresholds values were predictive of events in Asian patients. NFS >  − 1.455 predicted increased overall mortality in Japanese (HR: 12.87 (95% CI: 1.35–122.30), Table 2) [32] and Chinese (HR: 2.74 (95% CI: 1.67–4.50), Table 2) [33] participants followed-up for 4.6 and 6.6 years, respectively. In the Chinese study, a lower cut-off value (NFS >  − 1.836) is recommended to increase the prognostic accuracy of overall mortality in its population (sensitivity: 88.3%; specificity: 61.9% for the prediction of 6.6-year mortality, Table 2) [33]. An even lower threshold (NFS ≥  − 2.08) is set in a Korean study for optimal prognosis of overall and liver-specific mortality (HR: 1.43 (95% CI: 1.21–1.68), Table 2) [34]. Additionally, a non-specified threshold value for NFS predicted CVEs (Harrel’s c-index = 0.65, Table 1) [35], as well as LREs (HR: 1.78 (95% CI: 1.43–2.21), Table 2) [11], (Harrel’s c-index = 0.80 ± 0.02, Table 2) [35] within 30.9 and 81 months, respectively. Taken together, NFS can be considered a biomarker prognostic of decompensation events and mortality in patients with NASH cirrhosis. However, this score includes an invariable parameter such as age that will not change significantly during a typical trial duration of 6–12 months of treatment. In addition, patients with higher fibrosis tend to be significantly older than patients with milder fibrosis [29], and this could be reflected as a higher NFS value just as a function of age. Therefore, we consider the NFS a biomarker of rather low treatment monitoring utility (Table 3).

#### FIB-4

The Fibrosis-4 index (FIB-4) is a non-invasive score initially developed to stage liver disease in subjects with HIV-HCV co-infection [36] and with HCV infection alone [37]. Its formula includes age, AST, ALT, and platelet count. FIB-4 was later on applied as a marker of advanced fibrosis in NAFLD as well [38]. In the current review, a total of ten studies identified FIB-4 as prognostic biomarker of LREs and three of CVEs. Several threshold values are suggested for FIB-4 to be prognostic of CVEs, LREs, and/or mortality. FIB-4 > 2.67 is the most commonly used threshold and predicted CVEs (HR: 4.57 (95% CI: 1.60–12.98), Table 1) and mortality due to CVEs (HR: 2.68 (95% CI: 1.44–4.99), Table 1) occurring within 3.5–14.5 years [27, 28]. In addition, FIB-4 > 2.67 predicted LREs (HR: 13.1–14.6, Table 2) within 34.8–100 months follow-up [29, 31]. More in detail, FIB-4 > 2.67 predicted the development of HCC (HR: 3.66 (95% CI: 2.71–4.94), Table 2), liver transplantation (HR: 7.98 (95% CI: 4.62–13.79), Table 2) and end-stage liver disease (HR: 1.86 (95% CI: 1.68–2.05), Table 2) [39], overall mortality (HR: 2.49–10.52, Table 2) [29, 31, 39], increased hospital admissions (HR: 3.80 (95% CI: 2.79–5.19), Table 2), as well as duration of hospitalization (HR: 2.69 (95% CI: 1.92–3.78), Table 2) [29]. FIB-4 > 3.25 predicted LREs (HR: 6.33 (95% CI: 1.98–20.2), Table 2) [30] and the range 1.30–2.66 was used to predict overall mortality (HR: 1.13–1.46, Table 2) [28, 39] and end-stage liver disease (HR: 1.14 (95% CI: 1.07–1.22), Table 2) [39]. FIB-4 ≥ 1.30 predicted the development of HCC (HR: 8.46 (95% CI: 1.06–67.37), Table 2) over 2.5 years follow-up [40]. Similarly to what was the case for NFS, the lowest threshold (FIB-4 ≥ 1.22) was used in a study of Korean subjects where it predicted overall mortality (HR: 1.41 (95% CI: 1.18–1.68), Table 2) [34]. Finally, a non-specified threshold value for FIB-4 predicted CVEs (Harrel’s c-index = 0.60 ± 0.03, Table 1) [35] and LREs (Harrel’s c-index = 0.78 ± 0.03, Table 2) [35]. Taken together, FIB-4 can be considered a prognostic biomarker for decompensation events and mortality in patients with NASH cirrhosis. Although FIB-4 was also utilized as treatment monitoring biomarker [41], its formula contains age, which is an invariable parameter considering a typical trial duration. Hence, an observed treatment effect in FIB-4 is most likely attributable to changes in the values of the liver enzymes ALT and AST, as well as platelet count. We therefore consider FIB-4 of medium value as treatment monitoring biomarker (Table 3).

#### APRI

The AST to platelet ratio index (APRI) is a non-invasive score initially developed for the prediction of F3 and F4 in patients with chronic hepatitis C infection [42]. In the current review, one study investigates APRI as prognostic biomarker of CVEs, whereas four studies focus on LREs. Those studies identify baseline APRI > 1.5 as prognostic threshold for the outcomes considered [28–31]. In a large, multiethnic study including more than 11,000 patients, APRI > 1.5 predicted CVEs (HR: 2.53 (95% CI: 1.33–4.83), Table 1) [28]. In addition, APRI > 1.5 predicted LREs (HR: 5.02–20.90, Table 2) [29–31], increased overall mortality (HR: 3.1 (95% CI: 1.1–8.4), Table 2) [31], the occurrence of malignancies (HR: 4.94 (95% CI: 1.92–12.82), Table 2), and increased hospital admissions (HR: 2.49 (95% CI: 1.80–3.43), Table 2) as well as hospitalizations (HR: 2.90 (95% CI: 2.11–3.98), Table 2) [29]. Finally, a non-specified threshold value for APRI predicted LREs (HR: 1.88 (95% CI: 1.45–2.46), Table 2) [11]. Taken together, APRI can be considered a biomarker prognostic of decompensation events and mortality in patients with NASH cirrhosis. Given the formula of the APRI score, consisting of AST to platelet ratio, we consider this biomarker of high utility for treatment monitoring in patients with advanced liver disease (Table 3), and indeed evidence for the use of APRI as treatment monitoring biomarker exists [41]. It remains to be determined whether treatment-related changes in APRI associate with better outcomes.

#### BARD

The BARD (BMI, AST/ALT ratio, type 2 diabetes (T2D)) score was initially developed in a cohort of 823 NALFD patients of various ethnicities (Caucasian, Black, Hispanic, Asian Pacific Islander) considering BMI, AST/ALT ratio and T2D, where it showed a positive predictive value (PPV) = 43% and a negative predictive value (NPV) = 96% for the diagnosis of advanced fibrosis [43]. Because of its high NPV, this score seems to be more suited for ruling out the presence of fibrosis as to predict the occurrence of long-term outcomes, reflected by the low number of studies reporting on the prognostic ability of BARD. A non-specified threshold value for BARD predicted CVEs (Harrel’s c-index = 0.64 ± 0.04, Table 1), LREs (Harrel’s c-index = 0.73 ± 0.02, Table 2), HCC (Harrel’s c-index = 0.77 ± 0.03, Table 2), and extrahepatic cancer (Harrel’s c-index = 0.62 ± 0.04, Table 2) within 81 months [35]. BARD = 4 predicted the development of LREs (HR: 6.6 (95% CI: 1.4–31.1), Table 2) over a median of 104.8 months follow-up [31]. In the multicenter cohort study from Younes and colleagues, BARD was significantly outperformed by NFS and FIB-4 in the prognosis of long-term outcomes according to univariate cox proportional hazard models [35]. Given that the BARD formula includes T2D, i.e. invariable parameters considering a typical trial duration, we consider its treatment monitoring utility to be rather low (Table 3).

#### ELF™

The enhanced liver fibrosis (ELF™) test is a non-invasive test developed and patented by Siemens Healthineers that combines three serum biomarkers of fibrosis: hyaluronic acid (HA), tissue inhibitor of metalloproteinase-1 (TIMP-1), and amino-terminal peptide of procollagen III (PIIINP). The algorithm for its calculation was initially identified by Rosenberg and colleagues [44] and a population of patients with liver fibrosis of diverse etiology was used to determine threshold levels for the diagnosis of moderate liver fibrosis (≥ 7.7– < 9.8; sensitivity = 85%) and cirrhosis (≥ 11.3; specificity = 95%) [45]. In the USA, the ELF™ Test has been granted FDA authorization as prognostic risk assessment tool for patients with chronic liver disease by the FDA [46]. It can be used as prognostic marker in conjunction with other laboratory findings and clinical assessment tools in patients with advanced fibrosis due to NASH to assess the likelihood of progression to cirrhosis and liver-related clinical events. In a study of 475 Caucasian and Hispanic cirrhotic patients, ELF™ ≥ 11.27 predicted LREs (HR: 2.11 (95% CI: 1.53–2.90), Table 2) within 30.9 months follow-up [11]. Conversely, lower baseline ELF™ was associated with fibrosis regression [11]. The ELF™ test is widely used as treatment monitoring biomarker in recent clinical trials investigating new NASH treatments [41, 47–51]. In a phase IIa study of patients with compensated NASH cirrhosis, 16-week treatment with efruxifermin was associated with significant reduction of ELF score (− 0.4 efruxifermin vs. + 0.4 placebo; *p* = 0.0036) [52]. Hence, we consider the ELF™ test of high treatment monitoring utility as this parameter might be well suited to study treatment responses (Table 3), given that its constituents (i.e., HA, TIMP-1, PIIINP) are direct markers of liver fibrosis that are sensitive to change from baseline following treatment [51, 53].

#### HFS

The Hepamet Fibrosis Score (HFS) is a recently developed formula including age, sex, AST, albumin, homeostatic model assessment (HOMA), diabetes mellitus and platelet count [54]. Values of HFS ≥ 0.47 were used to identify advanced fibrosis (sensitivity: 35.2%; specificity: 97.2; PPV: 76.3%; NPV: 85.2%) and in doing so HFS demonstrated greater diagnostic accuracy compared to NFS and FIB-4 [54]. In the multicenter cohort study from Younes and colleagues, HFS was predictive of increased overall mortality in Caucasian subjects over a median follow-up of 81 months [35]. Given that the HFS contains invariable parameters such as age, sex, and diabetic status, we consider its treatment monitoring utility to be rather low (Table 3).

### Liver enzymes

Two studies were found where the liver enzymes measured were ALT and AST/√ALT. In a study of 42,282 American NAFLD patients of various ethnicities (Caucasian, Black, Hispanic), patients with liver steatosis + elevated ALT (> 40 IU/mL in men and > 31 IU/mL in women) were compared to patients with liver steatosis + normal ALT and those with no liver steatosis + normal ALT. Patients with liver steatosis + elevated ALT had a significantly increased incidence of HCC over a median follow-up of 8.4 years (HR: 4.35 (95% CI: 1.90–9.94), Table 2) [55]. In this group, 5-year and 8-year cumulative incidence rates of HCC were 1.0 and 1.4 per 1000 patients, respectively [55]. In another study including 7068 cirrhotic patients of various ethnicities the AST/√ALT was used as predictor of HCC development. Several ranges were tested and those with AST/√ALT > 6.45 (> 6.45–8.80, > 8.80–12.83, > 12.83) were predictive of HCC (HR > 1.99, Table 2) over a mean of 3.7 years follow-up [56]. Both liver enzymes are utilized as established treatment monitoring biomarkers. Nevertheless, it is also well accepted that ALT is not always elevated in patients with NASH [57]. Therefore, a reduction in ALT levels following treatment might not occur despite an effective therapy. For this reason, we consider the use of liver enzymes of rather low treatment monitoring utility in patients with NASH cirrhosis (Table 3).

### Alpha-fetoprotein

Alpha-fetoprotein is considered a diagnostic and prognostic biomarker of HCC, and high serum levels are associated with increased risk of HCC development and poor prognosis [58–60]. One study of Japanese participants found that values of alpha-fetoprotein ≥ 5 μg/L predicted HCC development (HR: 7.15 (95% CI: 1.44–35.6), Table 2) over a median follow-up of 4.2 years [61]. Given that alpha-fetoprotein is mainly used as biomarker for the screening and prognostic staging of HCC, we consider its treatment monitoring utility as rather low (Table 3).

### Platelet count

In the current review, a total of three studies were found investigating the platelet count as a prognostic biomarker of HCC, overall mortality, and liver-related mortality. In all three studies, a low platelet count was predictive of HCC development, although different thresholds are reported: < 150 × 10^3^/μL (HR: 3.67 (95% CI: 1.95–10.40), Table 2) [62]; < 146 × 10^3^/μL (HR > 2.18, Table 2) [56]; < 115 × 10^3^/μL (20.6% vs. 4.4% occurrence at 10 years in group with platelet count ≥ 115 × 10^3^/μL, Table 2) [63]. In addition, a platelet count < 115 × 10^3^/μL predicted lower overall survival (48.8% vs. 91.2%, Table 2) and lower liver-related survival (62.2% vs. 94.2%, Table 2) vs. ≥ 115 × 10^3^/μL [63]. Although the evidence suggests a low platelet count to be associated with bad prognosis, and although it might be affected by a successful therapy, it is unlikely that the platelet count could qualify as surrogate endpoint if employed as stand-alone biomarker. For this reason, we consider its treatment monitoring utility as rather low (Table 3).

### NLR and absolute lymphocyte count

The neutrophil-lymphocyte ratio (NLR) is an inflammatory marker relevant to tumor prognosis, as neutrophils tend to expand both in the tumor microenvironment and systemically and are associated with poor prognosis [64]. In addition, a reduced lymphocyte count can be symptomatic of reduced immune surveillance and lead to increased tumor growth and metastatic seeding [65]. Importantly, NLR has previously been found to predict the prognosis of patients with colorectal cancer [66], pancreatic cancer [67] and HCC [68] and has been associated with increased mortality in cirrhotic patients of various etiologies with HCC [69]. In the current review, one study was found linking NLR and absolute lymphocyte count to the prediction of HCC. NLR ≥ 3.09 predicted HCC development (HR: 1.43 (95% CI: 1.01–2.03), Table 2), whereas an absolute lymphocyte count ≥ 2.15 predicted lower HCC incidence (HR: 0.64 (95% CI: 0.43–0.94), Table 2) over 5.5 years follow-up [70]. Importantly, the authors of the study note that the NLR and lymphocyte count-associated risk of HCC development was independent of advanced fibrosis, as patients with mild fibrosis had the same risk to develop HCC as those with advanced fibrosis, provided their NLR and absolute lymphocyte count values were higher than the designated threshold. For this reason, the authors recommend using NLR and absolute lymphocyte counts as early markers of HCC rather than biomarkers for unidentified cirrhosis [70]. NRL and absolute lymphocyte count represent potentially interesting treatment monitoring biomarkers, as they reflect changes in immune cell dynamics that can be fluctuating according to the intensity of the immune response itself. For this reason, we consider NLR and absolute lymphocyte count of medium/high treatment monitoring utility, although limited to the monitoring of HCC only.

### sLOXL2

Lysyl oxidase (LOX) family members are extracellular copper-dependent enzymes playing an important role in ECM cross-linking and are involved in fibrosis progression in the liver [71–73] as well as in other organs [74, 75]. Therapeutic inhibition of LOX, Lysyl oxidase-like (LOXL) 1 or 2 induced fibrosis regression in animal models [71, 76, 77] but not in humans, as demonstrated by the failure of the selective LOXL2-blocking monoclonal antibody simtuzumab to reduce liver fibrosis in patients with HIV-HCV coinfection [78] and NASH [11, 79]. In a phase 2b clinical trial including 475 cirrhotic subjects, serum LOXL2 (sLOXL2) predicted the occurrence of LREs (HR: 1.02 (95% CI: 1.01–2.04), Table 2) over a median follow-up of 30.9 months [11]. Given the negative outcome of anti-LOXL2 treatment with simtuzumab in reducing hepatic fibrosis, it would be difficult to establish sLOXL2 as a reliable surrogate endpoint in future clinical trial. Therefore, we consider its treatment monitoring utility as rather low (Table 3).

### MiR-122

MiR-122 is a highly expressed micro-RNA in the liver [80] where it acts as tumor suppressor, as its loss or silencing is associated with tumorigenesis [81, 82] and its restoration in human HCC cells in vitro reversed their malignant phenotype [81, 83]. Previous studies have shown that the levels of circulating and hepatic miR-122 tend to decrease before fibrosis stage progression and HCC development [84–88]. The dynamics of circulating miR-122 in NAFLD, as well as whether it could serve as prognostic biomarker of LREs, was investigated in a small study including 81 Japanese patients with NAFLD followed-up for a median 7.6 years [87]. Two biopsied were taken from each patient (median time between biopsies: 2.9 years. Range 0.4–23.5 years) and concomitant to biopsy assessment miR-122 was measured in serum. Among those who developed HCC, miR-122 levels at second liver biopsy were significantly lower than in those patients without HCC. Patients with a miR-122 ratio < 0.5 (measurement at second biopsy relative to measurement at first biopsy) had higher cumulative rates of HCC compared to those with a miR-122 ratio ≥ 0.5 [87]. The data of this study are promising, as they are consistent with previous data linking decreased circulating miR-122 with HCC development. Nevertheless, confirmation in larger patients cohorts is needed in order to qualify miR-122 as treatment monitoring biomarker of HCC development in patients with NASH cirrhosis. For this reason, we consider miR-122 of medium treatment monitoring utility (Table 3).

### Imaging biomarkers

In addition to blood tests, imaging biomarkers are often utilized in clinical praxis as well as in clinical trials to assess liver health. In order to quantify liver fibrosis, imaging techniques are used to measure liver stiffness, which closely correlates with fibrosis stage [89] and serves as surrogate marker of fibrosis [90]. Furthermore, imaging biomarkers also have the advantage of providing an almost immediate result that can be shared with the patient.

### LSM

Liver stiffness measurement (LSM) includes a series of non-invasive techniques to measure liver elasticity (i.e., the resistance to deformation). LSM has been widely validated for the indirect staging of liver fibrosis [91, 92] and can be performed using ultrasound-based or magnetic resonance-based methods [93]. More recently, LSM has been demonstrated to be a valid prognostic marker being able to predict LREs, HCC development, and overall mortality. In the current review, one study on the prognostic utility of LSM in CVEs and five studies on the prognostic utility of LSM in LREs are outlined. LSM ≥ 2.97 kPa measured via magnetic resonance elastography (MRE) predicted coronary artery calcification (CAC) (odds ratio (OR): 3.53 (95% CI: 1.29–10.48), Table 1) over 19 months follow-up [94]. MRE-measured LSM ≥ 5 kPa predicted a series of outcomes (ascites, hepatic encephalopathy, varices, HCC, mortality) over at least 8 years follow-up (HR: 16.58 (95% CI: 2.90–94.79), Table 2) [95] and an increase in LSM > 19% at follow-up from baseline predicted decompensation (at least one between esophageal variceal bleeding, ascites, hepatic encephalopathy, jaundice) and overall mortality (HR: 19.04 (95% CI: 3.11–117), Table 2) [96]. A value of LSM ≥ 9.3 kPa measured with transient elastography (TE) predicted HCC (HR: 13.76 (95% CI: 2.83–66.95), Table 2) over 60.7 months follow-up [62]. A value of LSM > 12 kPa measured with Fibroscan^®^ predicted overall mortality (HR: 2.85 (95% CI: 1.65–4.92), Table 2) over 27 months follow-up time, as well as increased 5-year incidence of LREs (10.2% vs. 0.3% in group with LSM ≤ 12 kPa, Table 2) and death (13.8% vs. 3.4% in group with LSM ≤ 12 kPa, Table 2) [97]. Finally, a value of LSM ≥ 30.7 kPa measured with Fibroscan^®^ predicted LREs (HR: 10.52 (95% CI: 5.15–21.48), Table 2) over 16.2 months follow-up in Caucasian and Hispanic cirrhotic participants [98]. The accuracy of an imaging technique relies on the operator’s skills and depends on body composition, having lower accuracy in morbidly obese patients [99]. LSM is an established treatment monitoring biomarker, hence its utility can be considered high under well controlled conditions (e.g., fasted status of the patient, experienced investigator). For this reason, we consider LSM a biomarker of high treatment monitoring utility (Table 3).

### Combination of serum and imaging biomarkers

The combination of non-invasive tests in one score can yield improved accuracy in the diagnosis of fibrosis in NAFLD [100, 101]. Recently, a prospective study demonstrated that the combination of MRE-measured LSM ≥ 3.3 kPa and FIB-4 ≥ 1.6 (MEFIB index) provided a PPV of 97.1% for the diagnosis of severe fibrosis [101], but no data about the prognostic validity of such scores was available until recently. In the current review, one study reporting on the prognostic validity of the MEFIB index is outlined.

### MEFIB

The combination of MRE-measured LSM and FIB-4 gives rise to the MEFIB score [101]. Specifically, and according to regression models, a positive MEFIB score (defined as MRE-measured LSM ≥ 3.3 kPa and FIB-4 ≥ 1.6) was associated with a more than 21-fold higher risk of LREs development and overall mortality over a median follow-up ≥ 8 years in Caucasian and Hispanic NAFLD patients [95]. In addition, each 1-kPa increase in liver stiffness was associated with a more than twofold increased odds of hepatic decompensation or HCC. Interestingly, a positive MEFIB score was associated with a higher risk of LREs development and overall mortality compared to the risk associated with its singly-considered components (LSM between 3.65–5 kPa was associated with a 17-fold risk, whereas FIB-4 ≥ 1.6 was associated with a twofold risk) [95]. Although, to our knowledge, the MEFIB score has not yet been employed as treatment monitoring biomarker in clinical trials of therapeutics targeting NASH cirrhosis, the combination of imaging and serum biomarkers provided in this composite score could potentially serve for this scope. Further investigation is needed to validate the data of this study before informed judgments on the use of the MEFIB score in clinical trials can be taken. Given the increased risk of LREs associated with the MEFIB index as opposed to its single components (i.e., LSM and FIB-4), we consider the MEFIB score a biomarker of potentially high treatment monitoring utility (Table 3).

### Genomic biomarkers

#### PNPLA3 GG

The presence of the G allele in the patatin-like phospholipase domain containing 3 (PNPLA3) leads to the substitution of isoleucine with methionine at position 148 of the protein (I148M). The presence of this allele in homozygosis (PNPLA 3 GG) is associated with an increased risk of developing NAFLD and its progression to NASH, liver fibrosis, and cirrhosis, compared to the wild-type CC or the heterozygous CG phenotype [102]. One study investigating the prognostic ability of the PNPLA3 genotype in NAFLD was found. In this study, 238 Japanese patients with NAFLD were followed-up for a median of 6.1 years. Multivariate analysis revealed that the presence of the PNPLA3 GG genotype was a significant and independent risk factor for HCC development, especially when combined with advanced fibrosis [103]. In addition, Kaplan–Meier estimates revealed a significantly higher HCC incidence in patients with PNPLA3 GG genotype vs. PNPLA3 CC/CG genotype (10-year cumulative incidence: 30.7% vs. 2.7%) [103]. Although the PNPLA3 GG genotype cannot serve as a treatment monitoring biomarker as it is not influenceable by treatment, it constitutes a typical example of prognostic biomarker. Given the contribution of this gene variant to NAFLD progression and hepatocarcinogenesis, it holds potential for assisting in patient segmentation during clinical trials based on individual risk profiles.

## Conclusions

This systematic literature review provides a general overview of the prognostic biomarkers available for the prediction of CVEs, mortality, HCC, and other LREs in patients with NASH and advanced fibrosis. The review aims to guide clinical investigators in selecting non-invasive tests to be applied to NASH patients with advanced fibrosis as appropriate surrogate endpoints for clinical studies investigating novel NASH therapies. Identifying reliable prognostic biomarkers would aid in the development of novel therapies if the treatment-related change in these biomarkers would correlate with better outcomes. Bearing this in mind, we offer our perspective on the utility of the prognostic biomarkers identified in our search strategy as treatment monitoring biomarkers (summarized in Table 3). Nonetheless, our work has several limitations. First, not all studies in this review provided specific threshold values for the considered biomarkers, complicating the assignment of a single threshold value for predicting clinical outcomes. Our search revealed that threshold values for some biomarkers tend to vary, with fibrosis stage and ethnicity being key factors. Notably, when examining patients with F3/F4 fibrosis, we found that studies with predominantly Caucasian subjects reported higher average NFS values compared to those with predominantly Asian subjects (median NFS =  − 0.193 in Japanese subjects [32] vs. average NFS between 0.2 and 0.5 in studies of mainly Caucasian subjects [30, 31]). This observation supports the use of higher threshold values in Caucasian versus Asian subjects for predicting clinical outcomes and death. Lower threshold values in Asian NASH subjects also appear applicable for other serum biomarkers, such as FIB-4, BARD, APRI, and AST/ALT, as their commonly used threshold values exhibit lower sensitivities in this ethnic group [104]. The discrepancy in biomarker usage between Caucasian and Asian patients is unsurprising, given that the original studies establishing the commonly used threshold values primarily featured Caucasian subjects (NFS: 90% [26], FIB-4: 74% [36], APRI: 77% [42], BARD: 68% [43]). Additional reasons for this discrepancy may include differences in body fat percentage and BMI between Asian and Caucasian populations [105]. Indeed, Asian NAFLD subjects have been shown to have lower BMI than those from other ethnic backgrounds [106]. In a combined cohort study of minority groups living in the USA, Chinese-American and South-Asian individuals carried significantly greater risk of developing metabolic abnormalities compared to Caucasian subjects at similar BMIs in the overweight as well as in the normal weight ranges [107]. Metabolic abnormalities occurring in Caucasian patients at BMIs between 25 and 30 kg/m^2^ occurred in Chinese-American and South Asian subjects at BMIs between 19.6 and 24.5 kg/m^2^ [107]. This is consistent with the now generally recognized fact that obesity-related metabolic disorders begin at much lower levels of BMI in Asian patients [108]. Despite these limitations, a recent study indicates that the diagnostic performance of NFS, FIB-4, ELF, and LSM measured with vibration-controlled transient elastography was consistent across Hispanic White, non-Hispanic White, and Asian subjects. This finding suggests that these tests could be employed among these ethnic groups without requiring further calibration or cut-off adjustments [109]. However, not every study reports the relative frequency of ethnicities, which hinders the comparison of prognostic validity for a given biomarker across different studies. Furthermore, while we report the MEFIB score as a biomarker resulting from the combination of serum and imaging biomarkers, we also acknowledge the existence of the Fibroscan-AST (FAST) score as well, resulting from the combination of AST, LSM, and controlled attenuation parameter (CAP), the latter two measured with Fibroscan^®^. Our search strategy did not yield any results for the use of the FAST score as a prognostic biomarker in NASH patients with F3-F4 fibrosis; thus, to avoid potential bias, the FAST score was excluded from this review. However, a recent study identifies FAST > 0.35 as an independent predictor of LREs in NASH patients with HIV but without viral hepatitis [110]. Finally, some studies in this review feature relatively short follow-up periods, which may limit the assessment of long-term clinical outcomes in NAFLD patients, who typically have a median survival of over 10 years [111, 112]. Notably, a meta-analysis reveals that NASH patients who have compensated cirrhosis generally maintain this condition for approximately 4 years, accompanied by an annual 10% risk of progression to decompensation or death [113]. This data contributes an additional dimension to our biomarker prognosis research, considering the challenge of determining the disease stage of patients at the time of enrollment in interventional trials. Our systematic literature review indicates significant variability in follow-up durations. The majority of outcome trials do not establish a specific follow-up period, instead concluding this phase once a certain number of events have been documented. This study underscores the need for future research to incorporate direct comparisons of multiple biomarkers in their design, to overcome the limitations observed in the existing literature and provide a more robust evaluation of prediction accuracy. In conclusion, this systematic literature review shows that various non-invasive biomarkers can assist in risk stratification for patients with NAFLD/NASH and advanced fibrosis. Utilizing these biomarkers could prove advantageous for devising novel drug strategies specifically targeting advanced fibrosis. Several of these biomarkers show promise as treatment monitoring biomarkers in future clinical trials exploring cutting-edge therapeutic approaches against NASH cirrhosis.

## Data Availability

Not applicable.

## Abbreviations

ALT: Alanine aminotransferase
APRI: AST to platelet ratio index
AST: Aspartate aminotransferase
AUC: Area under the curve
BARD: BMI, AST/ALT ratio, type 2 diabetes
BEST: Biomarkers, EndpointS and other Tools
BMI: Body mass index
CAP: Controlled attenuation parameter
CI: Confidence interval
CVEs: Cardiovascular events
FIB-4: Fibrosis-4
HA: Hyaluronic acid
HBV: Hepatitis B virus
HCC: Hepatocellular carcinoma
HCV: Hepatitis C virus
HFS: Hepamet Fibrosis Score
HOMA: Homeostatic model assessment
HR: Hazard ratio
HVPG: Hepatic pressure venous gradient
LOX: Lysyl oxidase
LOXL: Lysyl oxidase-like
LREs: Liver-related events
LS: Liver stiffness
LSM: Liver stiffness measurement
MAFLD: Metabolic associated fatty liver disease
MELD: Model for end-stage liver disease
MeSH: Medical subject heading
MRE: Magnetic resonance elastography
NAFLD: Nonalcoholic fatty liver disease
NASH: Nonalcoholic steatohepatitis
NFS: NAFLD fibrosis score
NLR: Neutrophil to lymphocyte ratio
NPV: Negative predictive value
OR: Odds ratio
PIIINP: Amino-terminal peptide of procollagen III
PNLPA3: Patatin-like phospholipase domain containing 3
PPV: Positive predictive value
ROC: Receiver operating characteristic
T2D: Type 2 diabetes
TE: Transient elastography
TIMP-1: Tissue inhibitor of metalloproteinase-1
